# Renal actinomycosis with retroperitoneal abscess in a cirrhotic patient: A case report (CARE-complaint)

**DOI:** 10.1097/MD.0000000000018167

**Published:** 2019-12-10

**Authors:** Wei-Kai Liao, Shih-Che Huang, Sung-Yuan Hu, Che-An Tsai, Ren-Ching Wang

**Affiliations:** a Department of Emergency Medicine, Taichung Veterans General Hospital; b School of Medicine; c Institute of Medicine, Chung Shan Medical University; d Department of Nursing, College of Health, National Taichung University of Science and Technology; e Department of Nursing, Central Taiwan University of Science and Technology; f Division of Infectious Disease, Department of Internal Medicine, Taichung Veterans General Hospital; g Department of Pathology and Laboratory Medicine, Taichung Veterans General Hospital, Taichung, Taiwan.

**Keywords:** liver cirrhosis, renal actinomycosis, retroperitoneal abscess

## Abstract

**Rationale:**

Renal actinomycosis is a rare clinical infection, subacute to chronic presentation caused by the *Actinomyces* bacteria. *Actinomyces israelii* is diagnosed in the overpowering majority of reported cases. Abdominopelvic manifestation forms 10% to 20% of all actinomycosis, and may be misdiagnosed as either a malignancy or chronic inflammation due to the lower correct preoperative diagnostic rate (<10%).

**Patient concerns:**

A 38-year-old man with alcoholic liver cirrhosis experienced right flank pain, abdominal pain, and fever for 3 days. Leukocytosis, acute kidney injury, and impaired liver function were found. A computed tomographic scan demonstrated multiple renal cystic lesions, along with fluid accumulation at the right subphrenic and retroperitoneal spaces.

**Diagnoses:**

Renal actinomycosis was confirmed via cultures of both the abscess and nephrectomy specimen which grew *A israelii* and the pathological findings of multiple renal abscesses of actinomycosis with the characteristics of sulfur granules.

**Interventions:**

A nephrectomy was performed for an inadequate percutaneous drainage of renal abscess.

**Outcomes:**

A full course of antibiotics with intravenous penicillin G (3 million units every 4 hours) was prescribed for 2 weeks, followed by oral penicillin V given at a dose of 2 grams per day for 6 months at our out-patient facility.

**Lessons:**

A precise diagnosis of primary renal actinomycosis depends on any histopathological findings and/or cultures of specimens. A high dose of intravenous penicillin G is the first choice, followed by oral penicillin V, with the duration of each being dependent upon the individual condition.

## 1 Introduction

Renal actinomycosis (RA) is a rare subacute to chronic infection caused by the *Actinomyces* bacteria. *Actinomyces israelii* is diagnosed in the overpowering majority of reported cases.^[1–4]^ Abdominopelvic manifestation forms 10% to 20% of all actinomycosis cases.^[3]^ Primary RA is a rare clinical entity and may be misdiagnosed as either a malignancy or chronic inflammation due to the lower correct preoperative diagnosis rate (<10%).^[2,4,5]^ It has been reported in fewer than 20 cases in English medical publications since 1990.^[2,5–12]^The clinical presentations include pyelonephritis, renal or perirenal abscess, and masses.^[2]^ A computed tomography (CT) scan is a reliable diagnostic tool in offering adequate clues on the anatomical extent of RA.^[1–3]^ The diagnosis of primary RA is based on a histopathological evidence of biopsy, aspiration, or nephrectomy, or a microbiological culture of *Actinomyces*, or both.^[1,3,5]^ Clinical practice has shown that primary RA can be cured through antibiotics, with high doses of intravenous penicillin G for 1 to 2 months, followed by oral penicillin V for 6 to 12 months.^[1–4,6]^

## 2 Case report

**Figure 1:**
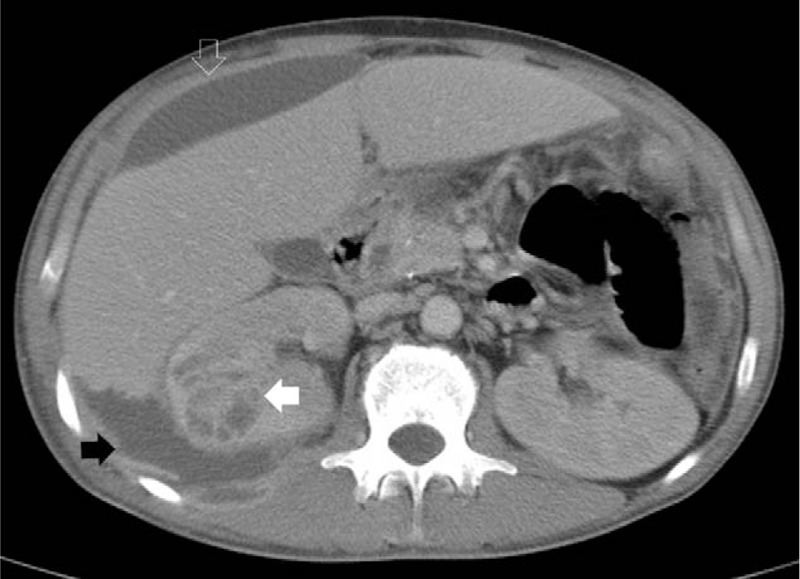
Abdominal computed tomography scan demonstrating multiple cystic renal abscesses with wall thickening (white arrow), irregular renal margin, fluid accumulation at the right subphrenic (white hollow arrow), and retroperitoneal (black arrow) spaces.

**Figure 2:**
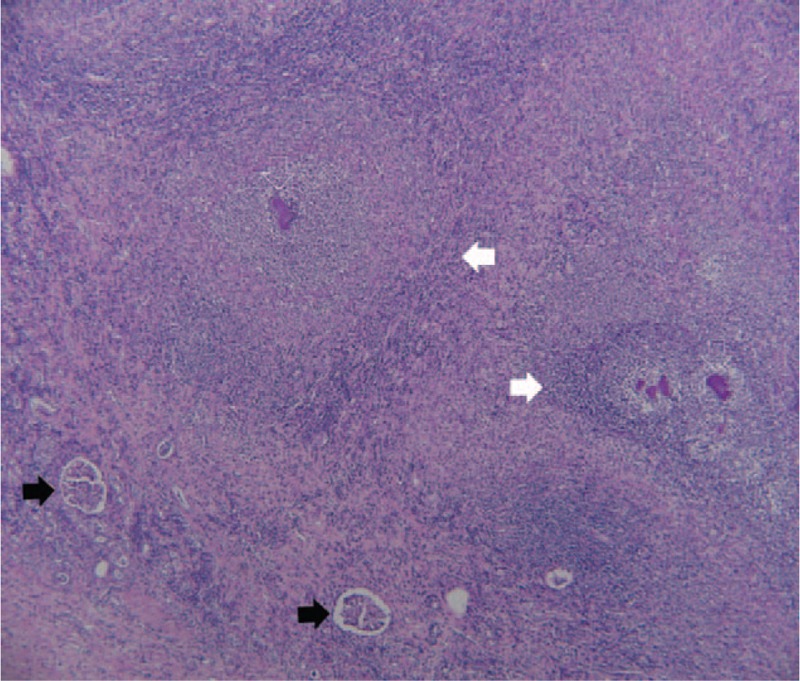
The pathological findings (hematoxylin and eosin stain, original magnification ×40) of the resected kidney revealing renal abscess (white arrows) containing filamentous bacterial colonies, that is, sulfur granules (white hollow arrows), with purulent infiltrations, and glomerulus (black arrows), compatible with the presentation of an *Actinomyces* infection in the kidney.

A 38-year-old Taiwanese man having a history of alcoholic liver cirrhosis, without regular medical follow-up, was admitted to the hospital. Right flank pain had occurred 10 days before this admission, and treatment was then prescribed for a muscle strain at a rural hospital. The pain, which radiated to the right upper abdomen, was accompanied with a fever, which developed for 3 days. The patient was then referred to our hospital for further analysis and management. Upon arrival to our hospital, his vital signs included a respiratory rate of 18 breaths/min, a heart rate of 122 beats/min, blood pressure of 120/72 mm Hg, and a body temperature of 39.5°C. A physical examination revealed mild pale conjunctiva, rapid regular heart beats, tenderness, and rebounding pain over the right upper quadrant of the abdomen, and also a knocking tenderness over the right flank region. Laboratory tests uncovered a white blood cell count (WBC) of 31,700/mm^3^, with segmented neutrophils at 96.6%, hemoglobin at 10.8 g/dL, platelet counts of 407 × 10^3^/mm^3^, blood urea nitrogen at 50 mg/dL, creatinine at 2.7 mg/dL, lactate at 16.3 mg/dL, albumin at 2.3 g/dL, glutamic-oxaloacetic transaminase at 122 U/L, glutamic-pyruvic transaminase at 49 U/L, lactate dehydrogenase at 486 U/L, alkaline phosphatase at 432 U/L, C-reactive protein at 40.4 mg/dL, blood glucose at 106 mg/dL, along with other test result levels which were unremarkable. A CT scan demonstrated fluid accumulation at the right subphrenic and retroperitoneal spaces, multiple cystic lesions within the right kidney, along with an irregular surface of the kidney adjacent to retroperitoneal fluid (Fig. 1). Purulent fluid was aspirated from the right subphrenic space for analysis, with the results indicating there was a WBC of 78,750/mm^3^, with an 85% level of segmented neutrophils. Percutaneous catheters were implanted for drainage of the right subphrenic and retroperitoneal abscesses using ceftriaxone at 2000 mg once daily. Six days later, an uncontrolled sepsis persisted due to the patient's failure to respond to medical treatment. Subsequently, a nephrectomy for the renal abscess was carried out involving debridement of the retroperitoneal abscess. Anaerobic cultures of the right subphrenic and retroperitoneal abscess grew both *A israelii* and *Bacteroides fragilis*. Aerobic cultures of the ascites and abscess uncovered no traces of bacteria. An anaerobic culture of the nephrectomy specimen yielded *A israelii*. Anaerobic, aerobic bacterial, and fungal cultures of the patient's blood and urine proved to be negative. Acid fast stain and cultures for tuberculosis were not found in either the urine or nephrectomy specimen. The pathologic results revealed multiple renal abscesses involving actinomycosis with the characteristics of sulfur granules (Fig. 2). A full course of antibiotics with intravenous penicillin G (3 million units every 4 hours) was prescribed for 2 weeks, followed by oral penicillin V given at a dose of 2 grams per day for 6 months at our out-patient facility.

### 2.1 Consent for publication

Written informed consent was obtained from the patient for publication of this case report and all accompanying images. Ethical approval was not required, but the patient's informed consent and agreement of images for publication were completed.

## 3 Discussion

*Actinomyces* are Gram-positive anaerobic bacteria that exist as normal flora in the oral cavity, tonsillar crypts, gastrointestinal, respiratory, and genitourinary tracts in humans. Actinomycosis is a rarely subacute to chronic clinical infection caused by the *Actinomyces* bacteria. *A israelii* is diagnosed in the overpowering majority (80%) of reported cases.^[1–4]^

Any tissue or organ in the human body can be infected with *Actinomyces*.^[3]^ Abdominopelvic manifestation forms 10% to 20% of all actinomycosis cases. RA may be classified as primary and secondary, and hematogenously spread from other infectious lesions.^[2,5,6]^ Primary RA is a rare clinical entity, with fewer than 20 cases being reported in English medical publications since 1990.^[2,5–12]^ The manifestations of primary RA include pyelonephritis, renal or perirenal abscess, and masses.^[2]^ Risk factors associated with the acquisition of actinomyces may involve diabetes mellitus, malignancy, immunosuppression (steroid, chemotherapy, and transplant), alcoholism, liver cirrhosis, and local tissue damage (which may involve trauma, surgery and irradiation).^[3]^ Primary RA is known to be easily misdiagnosed as a malignancy or another chronic inflammatory disease due to the lower correct preoperative diagnostic rate (<10%).^[2,4,6]^ Clinical presentation of primary RA includes nonspecific symptoms such as fever, weight loss, and pain. Laboratory data are nonspecific, including results regarding anemia, leukocytosis, increased erythrocyte sedimentation rate, raised high-sensitivity C-reactive protein, and elevated alkaline phosphatase in hepatic actinomycosis.^[3]^

A CT scan is a reliable imaging tool used for both locating lesions and providing sufficient information on the anatomical involvement of RA. This includes an enlarged kidney infiltrated with multiple cystic lesions or with the presence of an inflammatory process.^[1–3]^

A definitive diagnosis of RA is based upon the histopathological findings of a percutaneous biopsy, needle aspiration or nephrectomy, or a culture of *Actinomyces*, or a combination of such. Microscopically, sulphur granules are colonies of bacteria that appear as intertwined branching filament masses on a hematoxylin-eosin stain.^[1,3,5]^

Clinical practice has shown that actinomycosis can be treated through high doses of antibiotics, involving 18 to 24 million units a day of intravenous penicillin G for 1 to 2 months, followed by oral penicillin V at a dose of 2 to 4 g per day for 6 to 12 months, depending upon the location of infection site, severity of disease, and the patient's response to management. Additionally, β lactams, doxycycline, clindamycin, erythromycin, and clarithromycin are alternative drugs which are considered. β lactamase inhibitors provide additional coverage against potential β lactamase producers such as *Staphylococcus aureus* or Gram-negative anaerobic enterobacteria in colesional actinomycosis.^[1–4,6]^

## 4 Conclusions

In conclusion, primary RA is diagnostically challenging due to it being easily misdiagnosed as a malignancy or chronic inflammation. A clear-cut diagnosis depends upon histopathological findings and/or cultures of specimens. Depending upon each patient's susceptibility to antibiotics, the first choice is a high-dose of intravenous penicillin G, followed by oral penicillin V, with the duration of each treatment dependent upon the individual condition.

## Acknowledgments

Many thanks for performing the percutaneous drainage by the radiologist and the postoperative critical care by the team of intensive care unit. This study was approved by the Institutional Review Board of Taichung Veterans General Hospital (No. CE18102A).

## Author contributions

**Conceptualization:** Sung-Yuan Hu, Che-An Tsai.

**Data curation:** Wei-Kai Liao, Shih-Che Huang, Ren-Ching Wang, Che-An Tsai

**Formal analysis:** Wei-Kai Liao, Shih-Che Huang, Ren-Ching Wang.

**Investigation:** Wei-Kai Liao, Shih-Che Huang, Sung-Yuan Hu, Che-An Tsai, Ren-Ching Wang.

**Supervision:** Sung-Yuan Hu.

**Writing – original draft:** Wei-Kai Liao, Shih-Che Huang.

**Writing - review & editing:** Sung-Yuan Hu.

All authors contributed to the interdisciplinary interpretation of the clinical, radiological and histopathological findings, edited the manuscript for important intellectual content and approved the final version.
